# A Personalized Physical Activity Program With Activity Trackers and a Mobile Phone App for Patients With Metastatic Breast Cancer: Protocol for a Single-Arm Feasibility Trial

**DOI:** 10.2196/10487

**Published:** 2018-08-30

**Authors:** Lidia Delrieu, Olivia Pérol, Béatrice Fervers, Christine Friedenreich, Jeff Vallance, Olivia Febvey-Combes, David Pérol, Brice Canada, Eva Roitmann, Armelle Dufresne, Thomas Bachelot, Pierre-Etienne Heudel, Olivier Trédan, Marina Touillaud, Vincent Pialoux

**Affiliations:** ^1^ Department of Cancer and Environment Léon Bérard Cancer Center Lyon France; ^2^ Inter-University Laboratory of Human Movement Biology University Claude Bernard Lyon 1 University of Lyon Lyon France; ^3^ Inserm U1052 Cancer Research Center of Lyon Léon Bérard Cancer Center Lyon France; ^4^ Alberta Health Services Department of Cancer Epidemiology and Prevention Research CancerControl Alberta Calgary, AB Canada; ^5^ Departments of Oncology and Community Health Sciences Cumming School of Medicine University of Calgary Calgary, AB Canada; ^6^ Faculty of Health Disciplines Athabasca University Athabasca, AB Canada; ^7^ Department of Clinical Research and Innovation Léon Bérard Cancer Center Lyon France; ^8^ Laboratory on Vulnerabilities and Innovations in Sport University Claude Bernard Lyon 1 University of Lyon Villeurbanne France; ^9^ Digital Health Data and Studies Department Nokia Technologies Issy-Les-Moulineaux France; ^10^ Department of Medical Oncology Léon Bérard Cancer Center Lyon France; ^11^ Institut Universitaire de France Paris France

**Keywords:** metastatic breast cancer, physical activity, oxidative stress, activity trackers, feasibility

## Abstract

**Background:**

About 5% of breast cancer cases are metastatic at diagnosis, and 20%-30% of localized breast cancer cases become secondarily metastatic. Patients frequently report many detrimental symptoms related to metastasis and treatments. The physical, biological, psychological, and clinical benefits of physical activity during treatment in patients with localized breast cancer have been demonstrated; however, limited literature exists regarding physical activity and physical activity behavior change in patients with metastatic breast cancer.

**Objective:**

The primary objective of this study is to assess the feasibility of a 6-month physical activity intervention with activity trackers in patients with metastatic breast cancer (the Advanced stage Breast cancer and Lifestyle Exercise, ABLE Trial). Secondary objectives are to examine the effects of physical activity on physical, psychological, anthropometrics, clinical, and biological parameters.

**Methods:**

We plan to conduct a single-center, single-arm trial with 60 patients who are newly diagnosed with metastatic breast cancer. Patients will receive an unsupervised and personalized 6-month physical activity program that includes an activity tracker Nokia Go and is based on the physical activity recommendation. Patients will be encouraged to accumulate at least 150 minutes per week of moderate-to-vigorous intensity physical activity. Baseline and 6-month assessments will include anthropometric measures, functional tests (eg, 6-minute walk test and upper and lower limb strength), blood draws, patient-reported surveys (eg, quality of life and fatigue), and clinical markers of tumor progression (eg, Response Evaluation Criteria In Solid Tumors criteria).

**Results:**

Data collection occurred between October 2016 and January 2018, and the results are expected in August 2018.

**Conclusions:**

The ABLE Trial will be the first study to assess the feasibility and effectiveness of an unsupervised and personalized physical activity intervention performed under real-life conditions with activity trackers in patients with metastatic breast cancer.

**Trial Registration:**

ClinicalTrials.gov NCT03148886; https://clinicaltrials.gov/ct2/show/NCT03148886 (Accessed by WebCite at http://www.webcitation.org/71yabi0la)

**Registered Report Identifier:**

RR1-10.2196/10487

## Introduction

Breast cancer is the most common cancer among women worldwide with >1.6 million new cases diagnosed annually and 54,062 incident cases in France in 2015 [1]. About 5% of breast cancer cases are metastatic at diagnosis, and 20%-30% of localized breast cancer cases become secondarily metastatic [2-4]. Metastatic breast cancer is considered incurable, and treatments are proposed to improve the quality of life and overall survival.

Fatigue and reduced quality of life are frequent in patients with metastatic breast cancer related to the site of metastasis and cancer treatment [5,6]. Evidence from meta-analyses and systematic reviews in patients with localized breast cancer has demonstrated the benefits of physical activity on multiple health outcomes [7-12]. However, only five studies have focused on the investigation of physical activity interventions in patients with metastatic breast cancer [13-17] despite the need, desire, and ability of these patients to engage in physical activity [14,15,18]. Some studies have already investigated the association among metastatic cancer, fatigue, and physical activity; however, the results are mixed and warrant confirmation, specifically in patients with metastatic breast cancer [13-16].

In the cancer context, activity trackers with step pedometers are increasingly being used to measure physical activity and promote physical activity behaviors [19-21]. The benefits of mobile eHealth apps and pedometers on physical fitness, physical activity, and quality of life of patients with breast cancer have been reported [22]. These devices might motivate people to remain active and facilitate reaching a personal or recommended goal because of the feedback received in real time (eg, steps) [23-25]. In addition, activity trackers are often linked to a mobile app with a personal interface that provides a summary of the physical activity (ie, light, moderate, and vigorous intensity), sedentary time, and the number of steps accumulated per day, week, and month.

Reportedly, regular physical activity and fatigue can affect blood biomarker levels, including inflammatory markers [26] and oxidative stress [27]. Evidence from randomized controlled trials (RCTs) has indicated an effect of physical activity in patients with cancer on the levels of circulating growth factors and cytokines (eg, interleukin 6 and tumor necrosis factor alpha) [28,29] and suggests that physical activity might markedly alter the frequency and functional competence of immune cell subsets of the innate immune system (eg, neutrophils, monocytes, and natural killer cells) [30,31]. Concerning the oxidative stress, an excessive accumulation of oxidative stress in cells has been shown to induce marked cellular and molecular damages and likely plays an important role in carcinogenesis, tumor promotion, and breast cancer recurrence and metastasis [32-34]. Moreover, plasma antioxidant defenses seem to be lower in women with breast cancer [34]. On the contrary, physical activity programs have shown to decrease the oxidative stress in patients with chronic diseases other than cancer, in particular through the improvement of enzymatic antioxidant defenses [27,35].

The Advanced stage Breast cancer and Lifestyle Exercise (ABLE) Trial was designed to address the gaps in the current literature. The primary aim is to determine the feasibility of an unsupervised and personalized physical activity intervention in patients with metastatic breast cancer. The secondary aims are to investigate (1) how the physical activity intervention changes the total global physical activity, sedentary time, and physical fitness; (2) how the physical activity intervention changes patient-reported outcomes, including the quality of life and fatigue; (3) how the physical activity intervention changes patients’ anthropometric measurements and body composition; (4) the barriers and facilitators of the adherence to a physical activity program; and (5) whether the physical activity intervention affects the oxidative stress and inflammation as biomarkers of the tumor progression.

## Methods

### Study Design

The ABLE Trial is a single-arm trial that is being conducted in the Léon Bérard Comprehensive Cancer Center (Lyon, France). The study protocol was approved by the French ethics committee (*Comité de protection des personnes Sud-Est IV*), and the study database was reported to the National Commission for Data Protection and Liberties (CNIL; reference number: 1994192). The study is registered on ClinicalTrials.gov (NCT number: NCT03148886).

### Study Population

The inclusion criteria for this study are as follows: (1) female; (2) aged 18-78 years; (3) newly diagnosed with primary or secondary metastatic breast cancer histologically confirmed (ie, within the last 3 months) and treated in a cancer center by chemotherapy or radiotherapy or hormonal therapy or targeted therapy; (4) Eastern Cooperative Oncology Group Performance status <2; (5) able to speak and understand French and able to complete questionnaires and follow instructions in French; and (6) valid health insurance affiliation. For patients willing to participate in the study, confirmation from their treating oncologist of no contraindications to physical activity is required.

The exclusion criteria for this study are as follows: untreated brain metastases; pregnancy; and contraindications to physical activity (eg, uncontrolled hypertension, cardiac disease). All patients must sign an informed consent form.

### Recruitment

**Figure 1:**
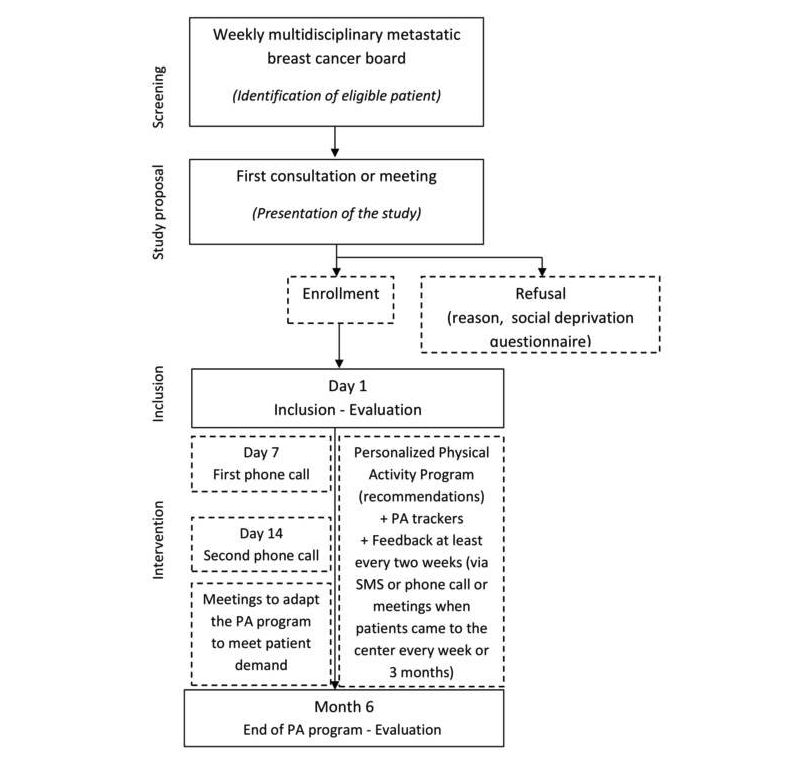
Participant flow chart for the Advanced stage Breast cancer Lifestyle and Exercise study, Lyon, France (PA: Physical activity).

Women are screened weekly during the center’s multidisciplinary metastatic breast cancer board meetings, as seen in Figure 1. After checking the inclusion and exclusion criteria, the study is directly proposed by an oncologist or radiotherapist to patients treated by chemotherapy or radiotherapy. As patients treated by hormonal therapy do not come to the center, they receive an information letter and study brochure through post and are contacted by telephone 1 week later to know whether they intend to participate. For all enrolled patients, a physician provides a certificate indicating no contraindications to physical activity, and an appointment is subsequently planned to sign an informed consent and proceed to the baseline evaluation (day one, D1).

### Intervention

#### Adapted Physical Activity Program

The evaluated intervention is a home-based, personalized physical activity program. The frequency, duration, and intensity of physical activity sessions are modulated depending on patients’ capacities.

Patients are encouraged to accumulate at least 150 minutes per week of moderate-to-vigorous intensity physical activity to maintain and improve health benefits. In addition, patients are asked to walk at least 30 minutes per day, increase their activities in their daily routine, and reduce their sedentary time. Several individual strategies are established with patients to attain their objectives (eg, using stairs whenever possible and walking for groceries or shopping). Moreover, meetings at the center with physical activity instructors are proposed depending on patients’ needs. Patients receive feedback at least monthly from their instructor. For patients undergoing chemotherapy, meetings take place during their day care visits at the cancer center. For patients with hormonal therapy treatment, meetings occur by phone or during a consultation with a physician in the center.

#### Activity Trackers

Activity trackers (Nokia Go wristband, Nokia France, Issy-les-Moulineaux, France) are given to all study participants. Patients are instructed to wear the device every day for the duration of the 6-month intervention. In addition, patients receive real-time feedback on their number of steps per day. Recommendations are to achieve 5000-10,000 steps a day depending on patients’ ability and comorbidities. For a patient who reaches 10,000 steps per day, we will advise her to maintain her goal. For a patient who is not able to achieve 5000 steps a day, we will gradually increase her objective. The goal is adjusted regularly throughout the 6-month intervention period. The instructor has access to the data pertaining to the number of steps per day and can modify the daily steps goal directly in the app or by a phone call to the patients during meetings.

Data are collected by regular transfer to the wearable activity tracker mobile phone app (Nokia Health Mate) available on a mobile phone or Tablet PC. Patients can use the mobile phone app to follow their number of steps per day represented by a graph. After receiving the wearable activity tracker, patients are called on days 7 and 14 to ensure the proper use of the device and answer any questions. Personalized objectives might be redefined to increase their daily physical activity. For patients without a mobile phone, data are collected during the 6-month intervention when visiting the hospital for a consultation every week or 2 weeks. Instructors might use the activity tracker interface to monitor change over time and to adapt to physical activity recommendations. At the end of the 6-month intervention, patients can keep the activity tracker to continue their efforts.

### Data Collection

Table 1 provides the complete data collection schedule in this study.

All assessments are recorded at the baseline and the end of the 6-month physical activity program (M6) by the instructor, as seen in Figure 1.

#### Demographic and Clinical Data

Demographic and clinical data, including date of birth, age at diagnosis, living situation, employment status, hormonal status, tumor histology, personal history of breast cancer, sites of metastases, and current treatment, are collected at the baseline. The Response Evaluation Criteria In Solid Tumors (RECIST) is used to assess the tumor progression between the diagnosis and the end of the physical activity program [36]. All clinical data are extracted from patients’ electronic medical records.

#### Body Composition and Anthropometrics

The standing height (cm), body weight (kg), waist (cm), and hip (cm) circumferences are measured using standardized procedures. The waist circumference is measured midway between the last floating rib and the iliac crest. The hip circumference is measured at the tip of the pubis. The body mass index is calculated as the body weight in kilograms divided by the square of the height in meters.

#### Physical Activity Fitness and Sedentary Behavior

Cardiorespiratory fitness is measured by evaluating the peak oxygen consumption during the 6-minute walk test (6MWT) [37,38]. Patients are asked to perform the maximum walk shuttle distance on 30-meter-long flat corridors in 6 minutes. During this test, the oxygen consumption, carbon dioxide production, heart rate, and oxygen arterial saturation are continuously recorded using a portable respiratory gas analyzer (MetaMax 3b; Cortex Biophysik, Leipzig, Germany). In addition, the perception of the difficulty during 6MWT is evaluated at the end of the test using the Borg Rating of Perceived Exertion questionnaire [39].

Then, prehensile and grip strength is measured using a hand dynamometry (Jamar Plus Digital Hand Dynamometer; Patterson Medical, Huthwaite, United Kingdom), which is a validated index of the elbow extension strength [40]. Moreover, patients are asked to squeeze the handgrip as strongly as possible for 5 seconds to obtain the maximal force. Of note, two measures are performed on each hand, and the best performance is registered.

The maximum isometric strength of quadriceps extension is measured using a back-leg dynamometer (DFS II Series Digital; Force Gauges Chatillon, Largo, FL, USA). Patients are asked to sit on a chair with the knee articulation at 90°, arms crossed on the chest, and the dynamometer attached to the ankle. At the signal of the instructor, patients must try to extend the leg as strongly as possible in 3 seconds. Only the dominant leg is tested twice, and the best performance is obtained.

The International Physical Activity Questionnaire (IPAQ) [41] is used to measure the self-reported physical activity. IPAQ (long form) is a validated self-administered physical activity questionnaire with 31 items and covers 4 activity domains, work-related physical activity, transportation physical activity, domestic physical activity, and recreational physical activity [41]. IPAQ gives specific scores in the metabolic equivalent of task (MET)-minutes/week for walking, moderate-intensity, and vigorous-intensity activity within each of the work, transportation, domestic chores and gardening (yard), and leisure-time domains. Questions are coded and converted in MET per minute and per week according to the Compendium of Physical Activities [42] by multiplying the number of METs by the duration and frequency of the activity. Then, the total physical activity score for each intensity is obtained by adding the score for this intensity in each domain and by adding the number of MET-minutes per week at each intensity.

**Table 1:** The data collection schedule for the Advanced stage Breast cancer Lifestyle and Exercise Trial.

Assessments		Time		
		Patients’ recruitment	Day 1: Baseline	Month 6: End of the study
**Clinical data (patient record)**				
	Date of birth		✓	
	Age at diagnosis		✓	
	Employment status		✓	✓
	Hormonal receptor status		✓	
	Personal history of breast cancer		✓	
	Metastasis localization		✓	✓
	Current treatment		✓	✓
	Tumor histology		✓	
	Disease progression (Response Evaluation Criteria In Solid Tumors)		✓	✓
Demographic data			✓	✓
Anthropometrics^a^			✓	✓
**Physical fitness**				
	6-minute Walk Test with oxygen consumption		✓	✓
	Upper limb strength: handgrip		✓	✓
	Maximum isometric strength of quadriceps extension		✓	✓
	International Physical Activity Questionnaire (long form)		✓	✓
	Sedentary behavior: Marshall Questionnaire		✓	✓
	Steps per day: Activity tracker^b^			✓
**Psychological questionnaires**				
	Quality of life: Cancer Quality Of Life Questionnaire, breast cancer module		✓	✓
	Fatigue: Piper Fatigue Scale		✓	✓
	Physical activity preferences, facilitators, and barriers	✓	✓	✓
	Incentive effect of activity tracker			✓
	Social deprivation: EPICES (Evaluation of Deprivation and Inequalities in Health Examination Centres) score	✓	✓	✓
**Other**				
	Reason for refusal	✓		

^a^Height, weight, waist-to-hip circumference, and body mass index.

^b^During 6 months.

Next, the global score of physical activity is divided into 3 categories commonly used by physical activity guidelines (<600 MET-minutes/week is equivalent to low physical activity; between 600 and 3000 MET-minutes/week is equivalent to moderate physical activity; and >3000 corresponds to high physical activity [43]). Furthermore, the sedentary time in minutes per week is obtained by adding the weekday sitting time in minutes for 5 weekdays, and the weekend sitting time in minutes for 2 days.

#### Patient-Reported Outcomes

The quality of life is measured with the European Organization for Research and Treatment of Cancer Quality Of Life Questionnaire (QLQ-C30) and its specific module for breast cancer (BR-23) [44]. QLQ-C30 is a 30-item self-administered questionnaire that evaluates 5 functioning domains (physical, role, emotional, cognitive, and social), a global quality of life domain, 3 symptom domains (pain, fatigue, and nausea), and 6 single items (dyspnea, insomnia, anorexia, diarrhea, constipation, and financial impact). Each item is associated with a score ranging from 0 to 100. For the functioning and global scales, a higher score corresponds to a better functioning level. The specific module for breast cancer (BR-23) gathers data about perceived body image, sexual functioning, sex enjoyment, arm symptoms, breast symptoms, and systemic therapy side effects.

Fatigue is assessed with the revised Piper Fatigue Scale [45,46], which is a 22-item self-reported questionnaire with 4 subscales, behavioral and severity, affective, sensory, and cognitive and mood. All these items together produce a score for total fatigue defining categories as follows: no fatigue (score=0), mild fatigue (score 1-3), moderate fatigue (score 4-6), and severe fatigue (score 7-10).

The social deprivation is assessed using the EPICES (Evaluation of Deprivation and Inequalities in Health Examination Centres) score [47,48]. The score is computed by adding each question coefficient to intercept whenever the answer is “yes.” The score ranges from 0 to 100 with the threshold for deprivation at 30, and higher scores indicate greater deprivation levels.

#### Determinants of Physical Activity

Preferences, facilitators, and barriers are evaluated with the translated version of a specific questionnaire developed by Vallance et al [49]; this questionnaire includes questions regarding the interest and willingness of patients with metastatic breast cancer to participate in a physical activity program designed for patients with metastatic breast cancer. In addition, it includes questions on their interest and preferences for physical activity counseling and specific aspects of these programs, including how, where, and when they would be interested in these physical activity programs as well as the type of intervention that would be most amenable for them.

#### Biological Data

A 7-mL blood sample is collected at the baseline (D1) and at the end of the study after 6 months (M6). The sample is centrifuged within half an hour after drawing and kept at 4°C before and during centrifugation. The plasma is distributed into cryotube aliquots of 1 mL and buffy coat in a single 1-mL cryotube; these cryotubes are frozen and stored at −80°C at the center and used for the analyses of oxidative stress and antioxidant biomarkers. Superoxide dismutase, catalase, glutathione peroxidase enzymatic activities, and markers of DNA oxidation (8-hydroxy-2'-deoxyguanosine), prostaglandins oxidation (8-Iso Prostaglandin F_2α__)_ are measured in the plasma [50]. Furthermore, levels of circulating growth factors, cytokines, neutrophils, monocytes, and natural killer cells are assessed as previously described [51].

### Statistical Analysis

#### Sample Size

The sample size was defined empirically to explore the feasibility of the program according to the enrollment potential in the study center. Because the main objective of the ABLE Trial was to assess the feasibility of a physical activity intervention in women with metastatic breast cancer, without major regard for the efficiency, no formal calculation was performed. Given that 200 patients are treated annually for metastatic breast cancer in the Center Léon Bérard and that 60% of patients are expected to survive for at least 6 months, with a projected acceptance rate of 50%, the pilot study will aim to recruit 60 patients within 9 months.

#### Statistical Methods

Patients’ characteristics will be described at D1 and M6 using the mean and SD for quantitative data, frequency, and percentage for qualitative data.

The primary outcome to assess the feasibility of the study will be the proportion of patients achieving the physical activity recommendations corresponding to 150 minutes per week of moderate physical activity evaluated by the IPAQ score during the last week of the study. In addition, we will estimate the proportion of patients who agreed to participate in the ABLE Trial among eligible patients. The reasons for refusal will be documented and described as well.

The secondary outcomes to assess will be the evolution of physical activity, anthropometric, physical fitness, psychological, and biological variables between the initiation (D1) and the completion (M6) of the physical activity intervention using Wilcoxon signed-rank tests. In addition, the level of physical activity according to biological, psychological, and clinical outcomes adjusted on potential confounders, including age, treatment, and number of visits, will be explored using multiple linear regressions. Moreover, the level of oxidative stress will be correlated to the RECIST criteria. Data on barriers and facilitators of the adherence to a physical activity program from D1 to M6 will be compared by Mac Nemar tests. All statistical analyses will be performed using SAS software (version 9.4. SAS Institute Inc, Cary, NC, USA).

## Results

The recruitment and enrollment in this single-arm feasibility trial started in October 2016. A follow-up was completed in January 2018. Data analyses began in February 2018 and will be completed in October 2018. All results are expected to be available by the end of 2018.

## Discussion

### Principal Findings

Given the beneficial effects of physical activity in localized breast cancer, the ABLE Trial is the first European study to propose a physical activity intervention for patients with metastatic cancer that will obtain preliminary data on biological, functional, psychological, and clinical outcomes and identify the determinants of physical activity. In addition, the use of wearable activity trackers in the ABLE Trial strengthens its novelty. Although wearable activity trackers are comparable to pedometers in some aspects, they are more effective for behavioral modification [52] because the Health Mate app and the wearable activity tracker provide more detailed data on the physical activity performed over time. These types of devices are emerging in the health care field and are being shown to help motivate people to increase their physical activity level and facilitate them to reach a personal or recommended goal because of the feedback received in real time (eg, steps) [23-25]. International recommendations for patients with cancer are to practice 30 minutes of moderate physical activity per day at least 5 times a week [53]. The number of steps per day is more easily comprehensible for individuals to achieve these physical activity recommendations. For adults with health impairment, 5000-7000 steps per day might be a more appropriate target than the target of 10,000 steps per day recommended for healthy people [54]. Indeed, walking is feasible at no charge and practiced by the majority of people. In the ABLE Trial, the recommended number of steps is individualized to each patient because research has suggested that a small goal is more effective and easier to achieve than a higher activity goal. In addition, it is important to increase these physical activity targets gradually [24].

The links between the level of physical activity and the biological mechanisms involved in the tumor progression have never been studied in patients with metastatic breast cancer. A study of the oxidative stress in this population might help identify potential new biomarkers associated with physical activity and tumor progression.

Of note, some limitations are acknowledged in the ABLE Trial. First, the sample size is limited but is sufficient to test the feasibility of the intervention. Second, the device used in the study has not been validated but uses the same algorithm as Nokia Pulse that has been validated by comparing this tracker with the OptoGait system for laboratory and ActivPAL for free-living conditions [20]. Finally, the test-retest reliability for Nokia Pulse was excellent with an intraclass correlation coefficient >0.90.

The results of this trial will provide quantitative and qualitative outcomes that will help design a future multicenter RCT on a physical training intervention in patients with metastatic breast cancer. Given the paucity of data in this population of patients and the potential for measurable health benefits to them, in many domains, this trial will provide new data that will be relevant in assessing the feasibility and acceptability of a larger-scale trial for this previously underinvestigated population.

### Ethics and Dissemination

The study protocol was approved by the French ethics committee (Comité de protection des personnes Sud-Est IV), and the study database was declared to the National Commission for Data Protection and Liberties (CNIL; reference number: 1994192).

## Abbreviations

6MWT: 6-minute walk test
ABLE: Advanced stage Breast cancer Lifestyle and Exercise
D1: day one
IPAQ: International Physical Activity Questionnaire
M6: end of 6 months
MET: metabolic equivalent of task
RCT: randomized controlled trial
SMS: Short Message Service
